# Supplementary material for: Causal Relationship between Aging and Anorexia Nervosa: A White-Matter-Microstructure-Mediated Mendelian Randomization Analysis
Source: Biomedicines. 2024 Aug 16;12(8):1874. doi: 10.3390/biomedicines12081874 (PMC11351342; doi:10.3390/biomedicines12081874)
Supplement: Supplementary file 1 [file biomedicines-12-01874-s001.zip › Supplementary Figure/Figure S8/Leaveoneout plot of MR analysis for TL to ubm-b-1454.pdf]

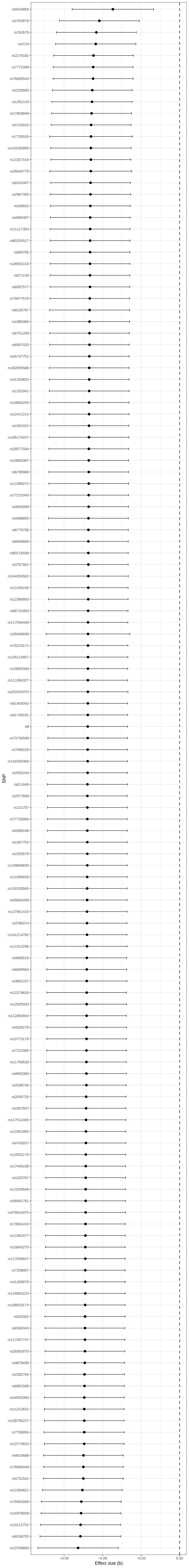
rs9419958
rs2763979
rs762679
rs4724
rs2276182
rs7772289
rs76065543
rs2230590
rs1291143
rs17803849
rs5742915
rs7705526
rs143190905
rs11557154
rs35640778
rs8102497
rs2967355
rs939916
rs4695407
rs11117354
rs80324517
rs869785
rs28502153
rs871134
rs6587577
rs79977579
rs8105767
rs1985369
rs6751209
rs6007020
rs55747751
rs182059586
rs41304832
rs1332941
rs10905255
rs12412214
rs1003322
rs185174247
rs28577594
rs10845387
rs6790988
rs11085072
rs77231040
rs9940099
rs4498805
rs6776756
rs6659669
rs80116508
rs3767952
rs144204502
rs11426156
rs12369950
rs66731853
rs117034449
rs35446936
rs76219171
rs145114957
rs10805346
rs111950327
rs202034370
rs61405042
rs61748181
All
rs73730598
rs7099229
rs142426306
rs2555104
rs611646
rs2977608
rs131797
rs77732866
rs9398196
rs1907702
rs2293579
rs139669835
rs11699829
rs150150565
rs45604339
rs137901416
rs3785074
rs141214782
rs11412296
rs9600019
rs6669563
rs3891167
rs11579626
rs12925933
rs112394943
rs4530278
rs10773176
rs7221585
rs11769630
rs9955360
rs2538745
rs2056726
rs1957937
rs117512405
rs12451892
rs4743037
rs12932179
rs17445108
rs1023767
rs13230646
rs56061761
rs376641875
rs73581419
rs11991877
rs10840270
rs117630647
rs7209057
rs41269079
rs116863223
rs188918174
rs932002
rs6590343
rs117407747
rs28363070
rs9878436
rs2282764
rs6881568
rs34550383
rs11212631
rs139795227
rs7790856
rs10774624
rs4616688
rs76666449
rs4731541
rs11584821
rs78491606
rs16978028
rs10112752
rs6536702
rs10768683
SNP
-0.09
-0.06
-0.03
0.00
Effect size (b)
